# An extraordinary presentation of mycobacterium avium cellulitis

**DOI:** 10.1016/j.jdcr.2025.11.016

**Published:** 2025-11-21

**Authors:** Nina Mehta, Anne Friedland, Jayson Miedema

**Affiliations:** a Department of Dermatology, University of North Carolina School of Medicine, Chapel Hill, North Carolina; b Physician, Department of Infectious Disease University of North Carolina School of Medicine, Chapel Hill, North Carolina; c Physician, Department of Dermatology University of North Carolina School of Medicine, Chapel Hill, North Carolina

**Keywords:** cellulitis, lung transplantation, Mycobacterium avium complex, nontuberculous Mycobacteria, panniculitis, skin

## Introduction

Nontuberculous mycobacterial skin infections typically present as nodules, ulcers, or draining sinuses and are most often caused by rapidly growing species such as *Mycobacterium abscessus* or *Mycobacterium fortuitum.*^1^ While *Mycobacterium chelonae* is most often implicated in localized mycobacteriosis, *Mycobacterium avium* complex (MAC) was identified in this case. In contrast, soft tissue infections due to MAC, a slow-growing organism, are rare and usually follow trauma or iatrogenic inoculation.^2^ Solid organ transplant recipients are at an increased risk for a variety of infectious panniculitides, including those due to fungi (eg, *Histoplasma*, *Cryptococcus*) and parasites (*Trypanosoma cruzi*), but MAC as a cause of cellulitis/panniculitis is uncommon. We present a highly unusual case of MAC presenting as cellulitis in a lung transplant recipient without known inoculation or environmental exposure. This case emphasizes the importance of considering atypical pathogens in immunocompromised patients with soft tissue inflammation unresponsive to standard therapy.

## Case report

A 74-year-old male with a history of bilateral lung transplantation 3 years prior presented with 1 day of progressive pain, erythema, and swelling of the right inner thigh. At presentation, he was receiving tacrolimus (dose: 0.5 mg AM/1 mg PM) and prednisone (dose: 7.5 mg daily) as immunosuppressive therapy. In this case, reduction of immunosuppression was not considered, and the regimen was maintained throughout his treatment due to stable graft function and risk of organ rejection. He denied prior injections or trauma to the area.

**Fig 1:**
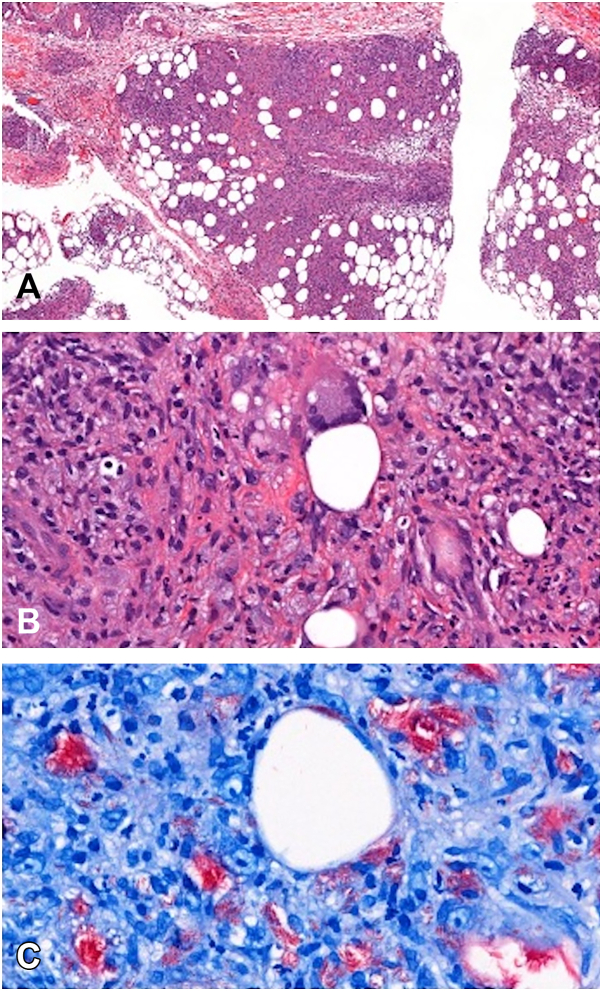
Clinical course. **A,** Initial presentation with warm, tender, erythematous indurated skin on right inner thigh. **B,** Worsening of the cellulitis 4 days after starting antibiotics. **C,** Relapse following discontinuation of therapy. **D,** Improvement observed 5 days after reinitiation of MAC-targeted treatment. *MAC*, *Mycobacterium avium* complex.

On examination, there was warmth, erythema, and tenderness without fluctuance. Computed tomography and ultrasound demonstrated subcutaneous edema but no abscess or gas. He was initially treated with broad-spectrum antibiotics for presumed bacterial cellulitis, but the erythematous area expanded and became more tender (Fig 1, *A*).

**Fig 2:**
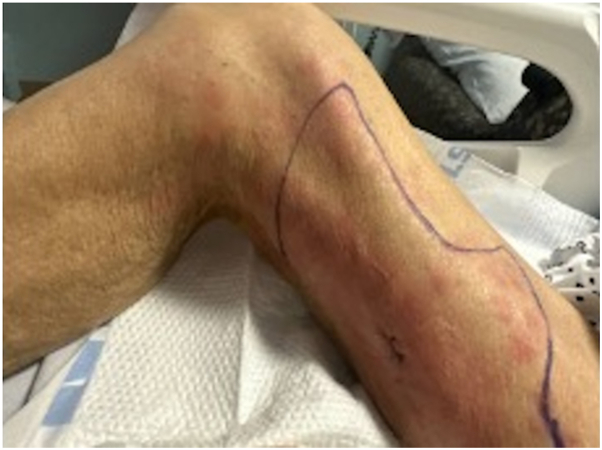
**A,** (Left) Low Power (H&E)–Punch biopsy showing dermal edema and panniculitis with sparse inflammation. **B,** (Middle) Medium Power (H&E)–Lobular panniculitis with granulomatous inflammation and histiocyte-rich infiltrate, including Langhans giant cells. **C,** (Right) High Power (Fite Stain)–Numerous acid-fast bacilli (AFB) visualized as bright red rods, confirming MAC infection. *H&E*, Hematoxylin and eosin; *MAC*, *Mycobacterium avium* complex.

A punch biopsy was obtained, revealing dermal edema with sparse inflammation and rare granulomas. Fite staining showed numerous acid-fast bacilli (AFB), and the AFB smear was 3+ (smeer collection site: lesion) (Fig 2, *C*). He was transitioned to linezolid, imipenem, azithromycin, and eravacycline with rapid improvement. Biopsy AFB cultures grew MAC, and biopsy fungal and bacterial cultures were negative. He was then transitioned to azithromycin, moxifloxacin, and ethambutol. Rifampin was not started due to interaction with his immunosuppressive medications. AFB blood cultures were negative, and there was no evidence of pulmonary infection on computed tomography chest or other evidence of disseminated infection.

The infection completely resolved, and therapy was discontinued after 2 months due to concerns about medication-related functional decline. Three weeks after stopping therapy, the patient returned with recurrent pain and erythema in the same location (Fig 1, *C*). Imaging again showed nonspecific soft tissue swelling without abscess. The family deferred treatment until repeat biopsy was obtained.

He underwent repeat biopsy, which again was AFB smear positive with cultures growing MAC and negative bacterial and fungal cultures, confirming relapsed infection. He restarted MAC therapy with azithromycin, ethambutol, and moxifloxacin and was consented for clofazimine. He developed prolongation of the corrected QT interval, requiring dose reduction of azithromycin from 500 mg daily to 250 mg daily. The cellulitis resolved again with MAC therapy (Fig 1, *D*).

His hospital course was complicated by delirium and failure to thrive. Although he was discharged on MAC therapy, it was discontinued approximately 6 weeks later due to medication intolerance and poor overall prognosis. He was transitioned to hospice care given ongoing failure to thrive and multiple comorbidities.

## Discussion

This case represents a rare cellulitic presentation of MAC infection in the absence of known trauma or inoculation or evidence of disseminated infection. Most reported cutaneous MAC cases involve indolent development of nodules or ulcers, often following surgical procedures or cosmetic injections.^1^^,^^2^ While immunocompromised patients are at an increased risk for atypical infections, a cellulitis-like presentation of MAC without a clear entry point or evidence of underlying disseminated infection is exceedingly rare.^2^

This case illustrates several diagnostic and management challenges. Clinically, the acuity of his presentation appeared consistent with a typical bacterial cellulitis. However, his lack of response to empiric antibiotics prompted a biopsy, which ultimately led to the diagnosis. In transplant recipients and other immunocompromised patients, clinicians should maintain a broad differential for soft tissue infections. Early biopsy with bacterial, fungal, and AFB staining and culture can be essential for diagnosis if the patients are not responding to empiric broad-spectrum antibiotics. Granulomatous inflammation and a positive AFB stain were key to identification in our patient. The acute presentation (severe cellulitis evolving over hours) is unusual for MAC, which is typically more indolent than rapidly growing mycobacteria.^3^

Second, MAC infections may require prolonged multidrug regimens, often for several months, and are best managed with infectious disease input. These complex, multidrug regimens can also be difficult to tolerate, as in our patient. In addition, the drug interactions between MAC therapies (particularly rifamycins) and immunosuppressive medications further complicate treatment in immunocompromised hosts. In this patient, initial improvement was followed by symptom recurrence after premature discontinuation of therapy, reinforcing the need for treatment adherence and close monitoring. Consideration was given to reducing the immunosuppressive regimen, but this was limited by the risk of lung transplant rejection.

Treatment of MAC pulmonary disease is outlined in consensus guidelines from the American Thoracic Society, European Respiratory Society, and Infectious Diseases Society of America, which recommend a macrolide, rifamycin, and ethambutol for at least 12 months after culture conversion. However, there are no standardized guidelines for extrapulmonary MAC. Management is often based on pulmonary protocols and adjusted based on clinical response.^4^ In this case, close coordination with infectious disease specialists and dermatology facilitated timely reinitiation of effective treatment. Given MAC’s slow-growing nature, therapy may need to continue for months, especially in immunosuppressed patients.^3^^,^^4^

Finally, the absence of classic findings such as nodules, ulcers, or draining sinuses can obscure the diagnosis. Clinicians should not dismiss atypical pathogens when confronted with cellulitis that fails to respond to initial broad-spectrum antibiotic therapy. A cellulitic presentation should not necessarily exclude atypical organisms.

## Conflicts of interest

None disclosed.
